# Sensitivity of Heterotrophic Bacteria in the Low-Salinity Water Areas and Estuary in Siak District toward Pathogenic Bacteria in Fish

**DOI:** 10.1155/2019/7456410

**Published:** 2019-06-10

**Authors:** F. Feliatra, Rizki Hamdani, Iesye Lukystyowati, Irvina Nurachmi

**Affiliations:** ^1^Marine Microbiology Laboratory Department of Marine Sciences, Fisheries and Marine Sciences Faculty, University of Riau, Pekanbaru, Riau, Indonesia; ^2^Parasite and Fish Diseases Laboratory Department of Aquaculture, Fisheries and Marine Sciences Faculty, University of Riau, Pekanbaru, Riau, Indonesia; ^3^Marine Chemistry Laboratory Department of Marine Sciences, Fisheries and Marine Sciences Faculty, University of Riau, Pekanbaru, Riau, Indonesia

## Abstract

The high rate of bacterial diseases in fishes and shrimps has lead scientists seek for natural antibiotic products that would act as a solution. An example of this product is the secondary metabolic products from heterotrophic bacteria. These bacteria could easily be found in many water regions and estuaries, including the Siak District, Riau, Indonesia. Therefore, this study aims at determining the ability of bacterial isolates in inhibiting the growth of pathogens (*Vibrio alginolyticus*, *Aeromonas hydrophila*, *and Pseudomonas* sp.). The research was conducted from June to September 2018. It actuates the type of heterotrophic bacteria in the sampling area using the PCR technique. The phylogenetic structure of bacterial isolates obtained during this study was assessed by nucleotide sequencing of the 16S rRNA gene. The antagonism test showed that bacteria had the ability to inhibit the growth of pathogens (*Vibrio alginolyticus*, *Aeromonas hydrophila*, and *Pseudomonas* sp.). The results showed that 25 pure bacterial isolates were obtained, in which 10 of those were carried out by DNA sequencing; hence, it could be used as antimicrobes. Based on the analysis of 16S rDNA, 10 isolates were identified: 6 were *Bacillus cereus* and 2 were *Pseudomonas aeruginosa* with homology levels ranging from 97 to 99%, while the remaining two were suspected as the new species of isolates. From the result, it could be concluded that heterotrophic bacteria are found to be better used as antipathogens against *Vibrio alginolitycus* than *hydrophila* and *Pseudomonas* sp.

## 1. Introduction

The involvement of microorganisms in the aquatic environment could not be ignored, as long as the heterotrophic bacteria have the ability to utilize organic substances as a nutritional source. The biogeochemical cycles in water also aids in decomposition to produce minerals and nutrients [1]. Heterotrophic bacteria are capable of utilizing organic and inorganic materials in their surrounding environment. It plays a major role in handling organic waste; therefore, the resulting effluent does not contaminate the environment. Over the years, the pollution of aquatic organic matter has continuously increased owing to the rise in industrial and domestic waste [2]. Heterotrophic bacteria are the most abundant organisms in the world with an abundance of around 10^6^/ml of seawater, making it ideal for studies on antimicrobes to be conducted on fishes. The tilapia fish suffer from numerous types of *Aeromonas* diseases, including veronii, sobria, and jandaei [3]. Meanwhile, the infectious diseases caused by motile aeromonads are one of the most common problems in freshwater aquaculture [4].

Marine bacteria including heterotrophic bacteria are capable of producing secondary metabolites. Unlike the primary metabolites, secondary metabolites are not needed for the survival of bacteria but are important for the adaptation process; besides, bacteria mainly produce secondary metabolites with antibiotic activity for survival purposes, and more recent studies show that these secondary metabolites also play a key role as molecules signaling [5, 6].

Some bacteria have benefits and some cause harm to living things. The secondary metabolite test performed on heterotrophic bacteria will produce antibiotic compounds that can inhibit the growth of pathogenic bacteria. Secondary metabolites of heterotrophic bacteria can be used as probiotics, where disease control strategies in fisheries are always carried out by using probiotics to give better results [7].

Many studies have been conducted to prevent losses caused by pathogenic bacteria, using antimicrobial compounds obtained from plants and animals or being produced by microbes commonly known as biopreservatives. For these reasons, this current study aims to determine the types and ability of heterotrophic bacterial isolates to inhibit the growth of pathogenic bacteria (*Vibrio alginolyticus, Aeromonas hydrophila*, *and Pseudomonas* sp.).

## 2. Methods and Materials

### 2.1. Time and Location of Research

**Figure 1:**
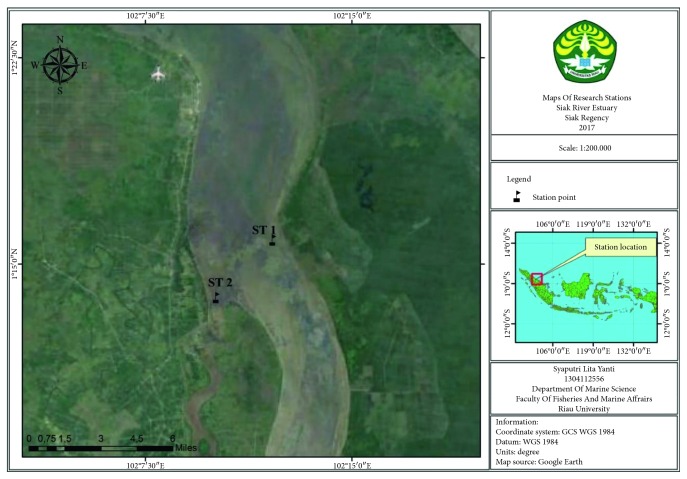
Research location map.

This research was conducted from June to September 2018. The sample was carried out at a stream of Siak River in Riau Province with two different stations (Figure 1). Station I is at the Padang Strait (salinity of 27 ppt), while station II is at the Siak River Estuary (10 ppt salinity). The sampling process was carried out at two stations. At each base, the three sampling points were determined with each at a distance of 50 meters. It comprises sea water and Van Dorn water sampler with a depth of about 10 m. The sample is inserted into a dark bottle and stored in the ice box at a temperature of 4°C.

### 2.2. Bacterial Isolation

About 1 ml samples of seawater were taken and put into a physiological solution (0.9% NaCl) to obtain 10^−1^ to 10^−6^ dilutions. At 10^−4^, 10^−5^, and 10^−6^ dilutions, as much as 1 mL was grown on the solid media *agar nutrient* (NA). It was incubated for 2 × 24 hours at a temperature of 28–30°C with the position of the cup inverted.

### 2.3. Identification of Bacteria

The bacterial isolates obtained were identified morphologically and biochemically. Morphological observations include the cell shape, colony color, size, and type. Biochemical tests include gram staining, catalase, methyl red, motility, indole, sulfide (H2S) citrate, and TSIA [8].

### 2.4. Sensitivity Analysis

The sensitivity analysis is carried out using the agar diffusion method [9, 10] by fighting pathogenic bacteria such as *Vibrio alginolyticus*, *Aeromonas hydrophila*, and *Pseudomonas* sp. One mL of purified pathogenic bacteria was extracted and planted into agar nutrient (AN) media, after which it was homogenously flattened. After condensation, paper discs dripped with antibiotic solution (Amoxan® 500°g) and were used as a positive control, with the NB medium as much as 0.5 *μ*l (negative controls) weighing 0.5 *μ*l isolates was inserted into it thrice. It was incubated at 28°C for 24 hours. The amount of antibacterial activity is determined by measuring the diameter of the clear zone around the discs.

### 2.5. Molecular Analysis

Molecular analysis was conducted to identify the bacteria by using 16S rRNA sequence. The DNA was extracted using the Presto™ Mini gDNA bacteria kit (GBB100 Geneaid). The DNA was then amplified using a pair primer of 24F: S′ AGA GTT TGA TGG CT 3′ and 1541R: S′ AAG GAG GTG ATC CAG CCG CA 3′. The PCR components included 1X PCR buffer, 0.1 *μ*M of dNTPs, 10 *μ*M of the forward primer (24F), 10 *μ*M of the reverse primer (1541R), 1 U of DreamTaq DNA polymerase (Thermo Scientific), 1 *μ*l of bacteria DNA, and Aqua Bidestilata, making up to a 50 *μ*l of PCR volume. The PCR program was performed as follows: pra-PCR at 95°C for 3 minutes, followed by 35 cycles that are performed at 95°C for 30 seconds, 50°C for 30 seconds, and 72°C for 1 minute and 30 seconds, and finally post-PCR at 72°C for 10 minutes.

The PCR products were migrated on 1% agarose gel containing 5 *μ*g/ml of ethidium bromide. The electrophoresis was conducted in the 1X TBE (Tris Borate EDTA) buffer on 50 volts for 45 minutes. After that, the bands were visualized on a UV transilluminator (Vilber Lourmat) and recorded using UV-filtered digital camera (Olympus SP 500-UZ).

Fourty *μ*l of PCR products and 30 *μ*l of primers were sent to PT. Genetika Science Indonesia for gel purification and sequencing at 1st BASE Malaysia.

### 2.6. Analysis of DNA Sequence

The DNA sequences that were obtained from sequencing using forward and reverse primers were put together to get DNA sequences for each bacterial sample. These sequences were then analyzed using the BLASTn program at http://www.ncbi.nih.nlm.gov/. The phylogenetic tree was reconstructed using MEGA6 software with the neighbor-joining method, Kimura-2-parameter model, and 1000 × bootstrap.

## 3. Results

From the isolation, 25 bacterial and 10 large antibacterial isolates were selected from the clear zone formation of the three test bacteria which was further tested for genotype. The 10 morphological observations of bacterial colonies showed slight significant difference in colonies with diameters of 0.5–1.5 cm. From the results of the observations, it can be seen that they possess milky-white and yellowish-white colonies (Table 1). They have a round shape with a slippery, wavy, and irregular coral edge. The surface is seen to have an embossed, flat, and convex surface.

Out of 10 isolates selected, 4 were from station 1 (salinity 27) and 6 from station 2 (salinity 10).

In general, the bacterial isolates obtained were 9 Gram-positive isolates and 1 Gram-negative isolate, all isolates were positive catalase, motile, negative indole, and able to ferment citrate. 1 isolate produces sulfide, whereas in the *methyl red* test, 9 isolates were negative and 1 isolate was positive. In the sugar test, 2 isolates were identified to be able to ferment glucose (Table 2).

Based on the sensitivity test in Table 3, it is found that the bacterial isolates have the ability to suppress the growth of pathogens characterized by the formation of inhibitory zones in the test media. The greater the diameter of the clear zone produced in the vitro test, the stronger the inhibitory power of an antimicrobial.

Observation shows that the inhibitory response for the growth of *V. alginolyticus* bacteria produced 6 heterotrophic bacterial isolates that belong to a strong inhibitory response, namely, isolates H2, H4, H8, H12, H15, and H16. The highest inhibitory value is found in H12 isolates with an average diameter of around 15.3 mm.

Furthermore, all bacterial isolates have the ability to inhibit the growth of *A hydrophila* which is 1.7 mm–7.2 mm. Five of them were categorized as moderate with the remaining 5 left tagged weak. From all isolates, H8 isolates obtained the highest inhibitory response with an average diameter of about 7.2 mm and included as a medium category.

The inhibitory response rate for the growth of *Pseudomonas* sp. showed that all bacterial isolates had a moderate inhibitory response ranging from 5.2 mm to 9.5 mm. The highest inhibitory value is found in H4 isolates with an average diameter of about 9.5 mm. Based on the antagonistic test, H8 and H12 isolates were found to have the highest inhibitory value against the three pathogenic bacteria (*V. alginolyticus*, *A. hydrophila*, and *Pseudomonas* sp.). The ability of bacterial isolates to inhibit the growth of pathogenic bacteria is a form of antagonistic activity carried out by producing antimicrobial compounds.

**Figure 2:**
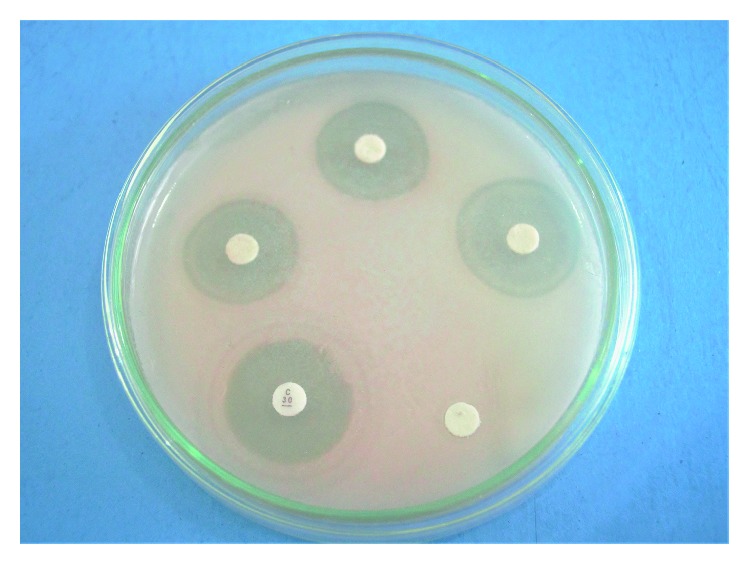
Sensitivity analysis results.

The difference in the ability of inhibitory power is caused by differences in the content of secondary metabolites possessed by each isolate that has first diffused into agar (Figure 2) so that the growth of the pathogenic bacteria becomes inhibited.

Based on the results of the BLAST analysis with regard to the GenBank through the website http://www.ncbi.nlm.nih.gov, it was shown that the ten isolates had a homology value of around 81–99% of the types of bacteria found in the GenBank database (Table 4).

From the analyzed isolates, 8 were found to have a homologous level of 97–99%. 6 of them were species of *Bacillus cereus* and 2 were species of *Pseudomonas aeruginosa*. Meanwhile, the remaining 2 left consists of homologs below 97%. They include H2 isolates which have 81% value against the bacterium *Bacillus* sp. strain DP5 and H10 isolates having a homology of around 91% against *Bacillus cereus* strain SN7 bacteria. It means that H2 and H10 are new species whose sequence of nitrogen bases has not been included in the GenBank database. Based on biochemical tests, these isolates are bacteria from the genus *Bacillus* with Gram-positive and motile characteristics. It comprises a round-shaped colony with a slippery and flat to arise or convex surface. It is also white or yellowish white in color and consists of the positive catalase, negative indole, and negative MR test.

## 4. Discussion

The ten isolates analyzed have varied inhibitory powers which are dependent on the response of the pathogenic bacteria. The highest response was obtained from *V. alginolyticus* bacteria and the lowest from *A. hydrophila* bacteria. This high and low inhibition depends on the secondary metabolites produced by the heterotrophic bacteria. According to Romanengko et al. [11], the biosynthesis of antimicrobial compounds plays an important role in the attachment process, as well as the target colonization until the competition obtains space and nutrients with other microbes. The ability to inhibit the growth of other bacteria is due to several factors such as the production of antibiotics, bacteriocins, siderophores, lysosomes, proteases, and hydrogen peroxide. It also affects the pH of the media by producing certain organic acids. This is in line with the research of Ravi et al. [8] which states that bacterial agents such as lactic acid can inhibit the growth of pathogens. It is because the antibacterial agents are able to reduce pH in order to inhibit the pathogenic bacteria.

**Figure 3:**
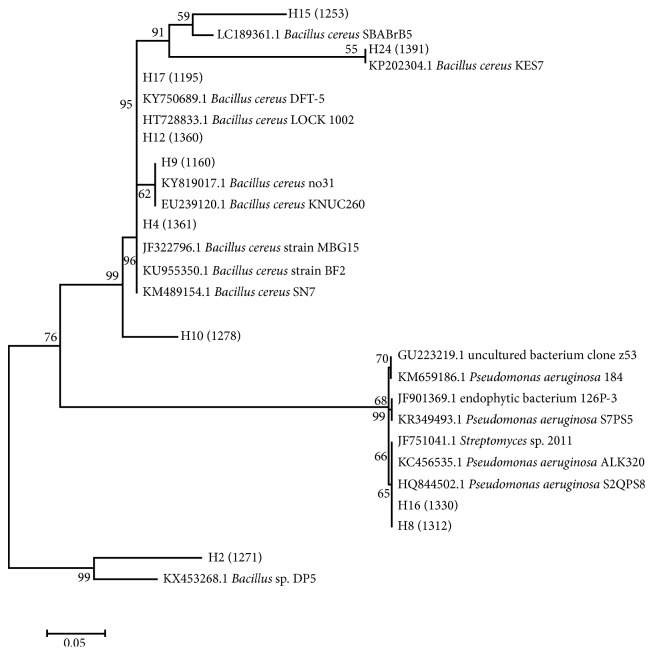
Ten isolates of the phylogenetic tree.

Six out of ten isolates were found which includes *Bacillus cereus* species (Figure 3). However, it had a close relationship with *Bacillus cereus*, for their homologs above 97%. *B. cereus* is a spore-forming bacterium belonging to the *Bacillaceae* family. Its spores are resistant to heat and radiation. Its characteristics are as follows: aerobic to facultative anaerobic and has a positive catalase. It is also included in the Gram-positive rod-shaped bacteria. This bacterium is one of the mesophilic organisms [12–14]. According to Verschuere et al. [15] and Das et al. [16], it was proved that *B. cereus* bacteria can produce antimicrobial compounds and can inhibit the pathogenic bacteria, namely, *V. alginolyticus, A. hydrophila*, and *Pseudomonas* sp. It is characterized by the formation of a clear zone in the antagonistic test.

This is owing to its ability to produce antibiotic compounds, a collection of chemicals produced by microorganisms including fungi and bacteria which have the function of inhibiting growth or killing other microorganisms. According to Nishijima et al. [17], *Bacillus* species produced at least 66 different types of antibiotics.

This bacterium is also often used in research related to probiotics. In general, the role of probiotics in aquatic environments includes maintaining pH balance during the day and night, accelerating the process of decomposition of waste, and eliminating toxic gases. *B. cereus* is widely used as a probiotic in aquaculture taken from the digestive tract [18] and has an antimicrobial substance called bacteriocin.

According to Drider et al. [19], bacteriocin is an antimicrobial polypeptide compound synthesized in ribosomes by Gram-positive or negative bacteria. It is not a toxic material, but a protein compound degraded by proteolytic enzymes. Bacteriocins are stable against a wide pH and temperature. Umoro [20] states that its compounds are produced by different bacteria in each type. In the type of *B. cereus*, Cerein GN105, Cerein 7A, and Cerein 7B were produced.

The main habitat of *B. cereus* is in the food and digestive tract. These bacteria can also be attached to shoes, clothing, and workers' skin and can spread through air or dust. Guinebretière et al. [21] stated that this type of bacteria is found not only in soil, water, and fermented foods but also in coastal waters.

H8 and H16 isolates were the two isolates analyzed which are included in the *Pseudomonas aeroginosa* species. They have 99% homology similarity with *P. aeruginosa* ALK320 strain and *P*. *aeruginosa* S2QPS8 strain. It means that the homology level is similar to the species level. It is supported by biochemical tests which show that H16 isolates are Gram negative, catalase positive, motile, and unable to ferment sugar. The statement is in line with Selezska et al. [22] analysis, which showed that *Pseudomonas* bacteria themselves have characteristics such as Gram-negative, rods or cocci, obligate aerobes, and motile having polar flagellum. These bacteria included positive oxidase and positive catalase and no fermenter. This is corroborated by Janda and Abbott [23] who stated that if the homology has a percentage approaching 100% or above 97%, it can be confirmed as the same species, but conversely if the homology is smaller than 97%, it is likely that the isolate is a new species.

*P. aeruginosa* is often identified with the pathogenic bacteria because in some cases, this bacterium can affect its host. Toward humans, these bacteria cause immunocompromised and cystic fibrosis diseases [24, 25]. Based on these characteristics, many of these bacteria are used in agriculture as agents of plant disease control. According to Mansoor et al. [26], based on the in vitro test, the application of *P. aeruginosa* can inhibit the growth of *Macrophomina phaseolina*, *Rhizoctonia solani*, and *Fusarium oxysporum* by producing inhibitory zones of 2, 6, and 10 mm, respectively. It explains that *P. aeruginosa* bacteria have the antibacterial properties against certain microbes.

This statement was emphasized by Yasmin et al. [27] who stated that *P. aeruginosa* Z5 significantly reduces the incidence of disease by suppressing the growth of *F. oxysporum* (the agent that causes cotton seedling disease). In addition, Azadeh and Meon [28] stated that *P. aeruginosa* strains of UPM P3 have the potential to suppress the pathogen of *Ganoderma boninense*, the cause of stem rot in basal stem rot (BSR) in oil palm.

Furthermore, *P. aeruginosa* also produces pyoverdine and salicylic acid which are effective against *Peronospora tabacina* in tobacco plants, *Alternaria solani* in tomatoes, and *Pseudoperonospora cubensis* in cucumbers [29]. Besides producing pyoverdine and salicylic acid, it also produces bacteriocins called pyocin [30]. It is also used to fight and kill other types of bacteria. According to Briand and Baysse [31], pyocins of *P. aeruginosa* are located on the chromosome and also divided into three types, i.e., pyocin R, pyocin F, and pyocin S.

The last two isolates, H2 and H10, which had the highest homologies on GenBank by 81% and 91% are considered to be the new types of isolates not previously identified. Both isolates have the ability to be antipathogenic bacteria in fish and shrimp. According to Hagström et al. [32], the isolates that have more than 97% homology equations can be represented at the same level of species. Then, the homology equation between 93% and 97% can represent the identity at the genus level but differs at the species level. However, if under 93%, it is likely that it is a new species whose sequence of nitrogen bases has not been included in the GenBank database.

## 5. Conclusion

Based on the concluded research, 10 heterotrophic isolates were able to inhibit the growth of pathogenic bacteria (*Vibrio alginolyticus, Aeromonas hydrophila*, *and Pseudomonas* sp.). H8 isolates from station 1 and H12 from 2 were found to be the best isolates capable of inhibiting the growth of all three pathogenic bacteria. All isolates have the strong ability to prohibit the growth of *V alginolyticus* bacteria and weak ability against *Aeromonas* sp. The identification results using 16S rRNA analysis revealed that among 10 isolates identified, 6 of them belonged to the *Bacillus cereus* family and 2 to the *Pseudomonas aeruginosa* with homology levels ranging from 97 to 99%. The last 2 are considered to be the new bacteria, which are H2 and H10 with 81% and 91% homology levels. The heterotrophic bacterial isolates were better used as antipathogens for the *Vibrio* sp. bacteria than for hydrophila and *Pseudomonas* sp.

## Figures and Tables

**Table 1:** Morphology of heterotrophic bacterial isolates.

Station	Isolate name	Diameter (cm)	Colony color	Colony shape	Edges	Surface
1	H2	0.6	Milky white	Round	Slippery	Embossed
1	H4	1.5	Milky white	Round	Slippery	Embossed
1	H8	0.5	Milky white	Round	Slippery	Embossed
1	H9	0.5	Milky white	Round	Slippery	Embossed
2	H10	0.8	Yellowish white	Round	Slippery	Flat
2	H12	1	Yellowish white	Round	Wavy	Flat
2	H15	0.8	Milky white	Round with a coral edge	Irregular	Convex
2	H16	0.8	Milky white	Round	Slippery	Flat
2	H17	1	Milky white	Round	Slippery	Embossed
2	H24	0.7	Milky white	Round	Slippery	Embossed

**Table 2:** Biochemical test of each isolate.

Biochemical test	Sample code									
	H2	H4	H8	H9	H10	H12	H15	H16	H17	H24
Gram	+	+	+	+	+	+	+	−	+	+
Catalase	+	+	+	+	+	+	+	+	+	+
Motility	+	+	+	+	+	+	+	+	+	+
Indole	−	−	−	−	−	−	−	−	−	−
H_2_S	−	−	−	−	+	−	−	−	−	−
MR	−	−	−	−	−	−	−	−	+	−
Citrate	+	+	+	+	+	+	+	+	+	+
TSIA										
T	M	M	M	M	M	M	M	M	M	M
M_1_	M	M	M	M	M	M	K	M	M	K
Sugar test										
Glucose	−	−	−	−	−	−	+	−	−	+
Lactose	−	−	−	−	−	−	−	−	−	−
Sucrose	−	−	−	−	−	−	−	−	−	−

MR: methyl red test; −: negative; +: positive; T: upright; M_1_: tilt; K: yellow; M: red.

**Table 3:** Antagonism test results of bacterial isolates against pathogenic bacteria.

Bacterial isolates test	Diameter of the inhibition zone (mm)														
	*V. alginolyticus*					*A. hydrophila*					*Pseudomonas* sp.				
	(+)	*U*_1_	*U*_2_	*U*_3_	*R* (mm)	(+)	*U*_1_	*U*_2_	*U*_3_	*R* (mm)	(+)	*U*_1_	*U*_2_	*U*_3_	*R* (mm)
H2	6	14	12	13	13	4	7	6.5	6.5	6.7	5	7	6.5	7.5	7
H4	3.5	11	12.5	14.5	12.6	3.5	1	2	2	1.7	3	5	12.5	11	9.5
H8	7.5	12	11	11.5	11.5	7	11	6	4.5	7.2	6	6	10.5	11	9.2
H9	9	0	3	4	2.3	6.5	5.5	5	4.5	5	2	6.5	7	5.5	6.3
H10	8	5	5	6	5.3	5.5	7	6	5.5	6.2	7	7.5	8	9.5	8.3
H12	6	15	16.5	14.5	15.3	10	5.5	3.5	4	4.3	2	11.5	6	7	8.2
H15	7	13	9.5	9	10.5	7.5	4	2	3.5	3.2	7	6	4	5.5	5.2
H16	3.5	8	13	9	10	2	5.5	5	7	5.8	3	9.5	6	7	7.5
H17	4	11	9	9	9.7	8	7	4.5	5	5.5	6	7.5	11	6.5	8.3
H24	3	0	6	1	2.3	5	2.5	4.5	1.5	2.8	1	5	6	9.5	6.8

*U* = repetition; *R* = average.

**Table 4:** BLAST results (Basic Local Alignment Search Tool).

Isolate	Species	Strain	Access code	Homology (%)
H2	*Unculture bacteria 1*	DP5	KX453268.1	81
H4	*Bacillus cereus*	BF2	JF322796.1	98
H8	*Pseudomonas aeruginosa*	ALK320	KC456535.1	99
H9	*Bacillus cereus*	no31	KY819017.1	99
H10	*Unculture bacteria 2*	SN7	KM489154.1	91
H12	*Bacillus cereus*	LOCK 1002	KT728833.1	99
H15	*Bacillus cereus*	SBABrB5	LC189361.1	98
H16	*Pseudomonas aeruginosa*	S2QPS8	HQ844502.1	99
H17	*Bacillus cereus*	DFT-5	KY750689.1	99
H24	*Bacillus cereus*	KES7	KP202304.1	97

## Data Availability

The data used to support the findings of this study are available from the corresponding author upon request.

## Supplementary Materials

Supplementary Materials. Table 1: results of the measurement of the quality of the waters. Figure 1: Elektropherogram DNA bacteria. Table 2: FASTA DNA sequence of each isolate.
