# Supplementary material for: Sensitivity of Heterotrophic Bacteria in the Low-Salinity Water Areas and Estuary in Siak District toward Pathogenic Bacteria in Fish
Source: Int J Microbiol. 2019 Jun 10;2019:7456410. doi: 10.1155/2019/7456410 (PMC6590592; doi:10.1155/2019/7456410)
Supplement: Supplementary Materials. — Table 1: results of the measurement of the quality of the waters. Figure 1: Elektropherogram DNA bacteria. Table 2: FASTA DNA sequence of each isolate. [file 7456410.f1.pdf]

Table 1. The Results Of The Measurement Of The Quality Of The Waters

station	Kordinat	pH	Salinity (ppt)	brightness (cm)	temp (^{o}C)	current (m/s)	OD (mg/L)
Stasiun 1 N<br>E	01° 06’41,6’’<br>102°12’19,2’’	6,5	27	23,5	29	0,5	6,2
Stasiun 2 N<br>E	01^{0}12’ 26’’<br>102^{0}09’ 53,7’’	6	10	15	28	0,3	5,24

OD Oxygen dissout

Figure 1. Elektroforegram DNA Bacteria

- Isolat H2

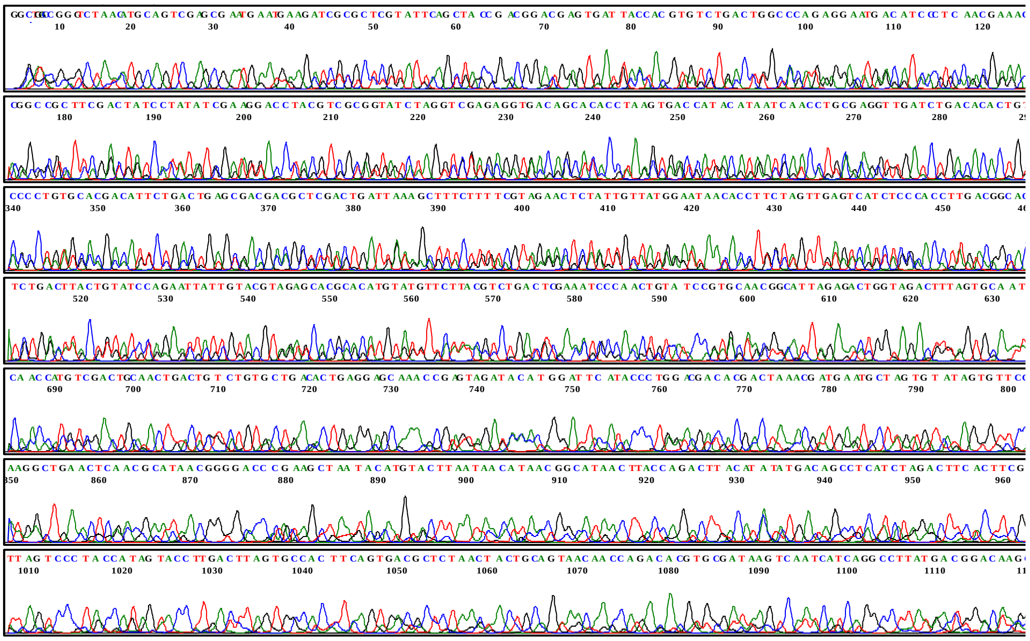

- Isolat H4

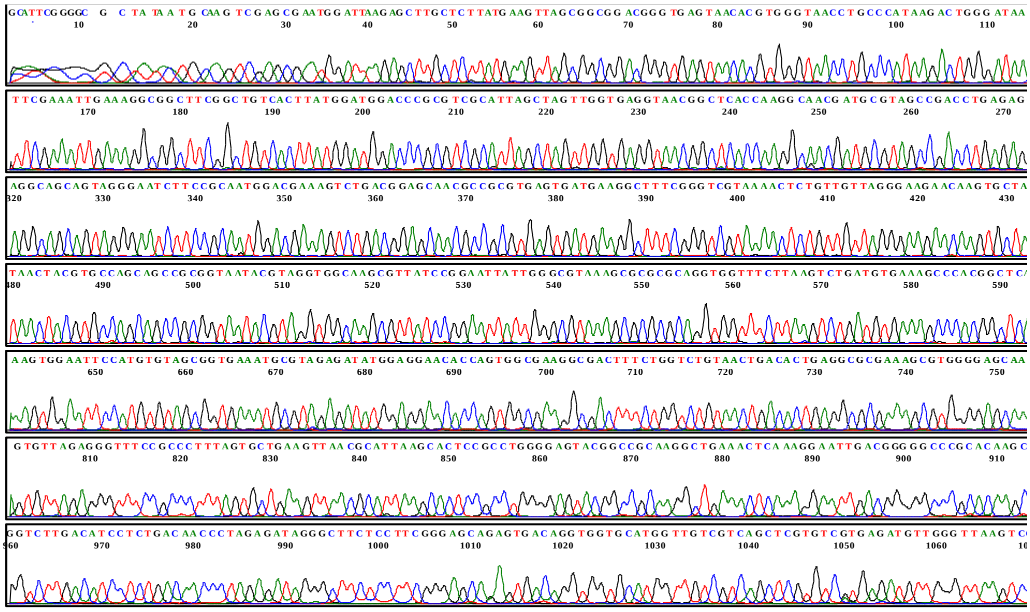

- Isolat H8

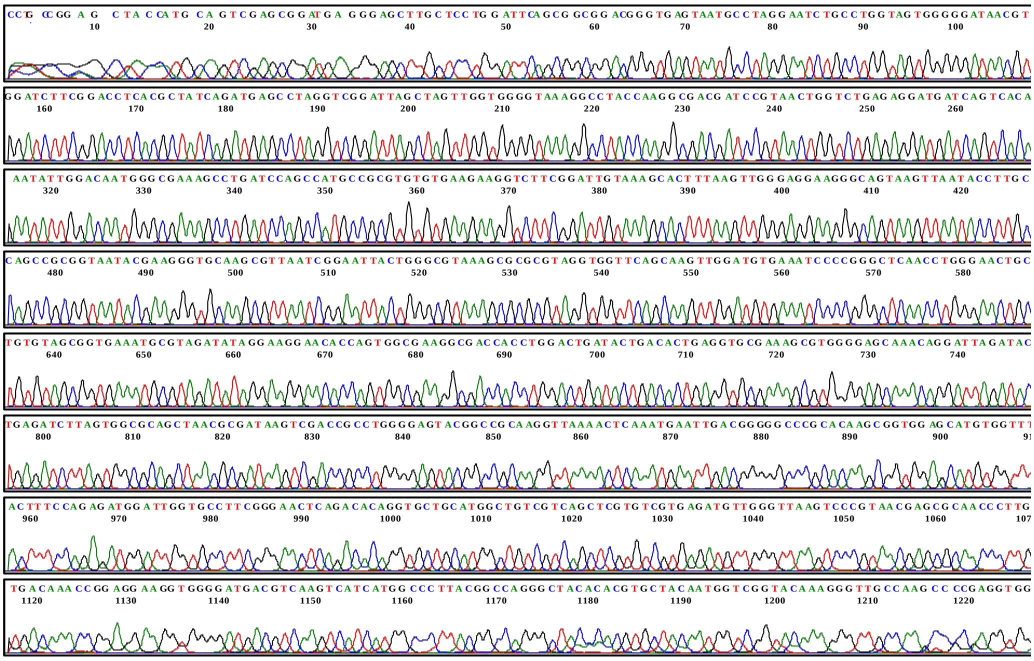

- Isolat H9

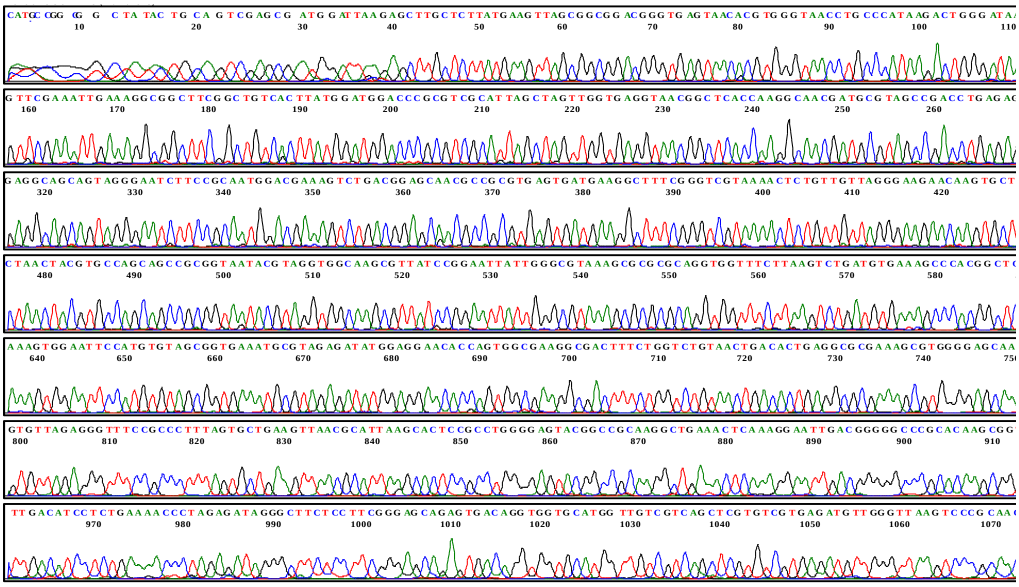

- H10

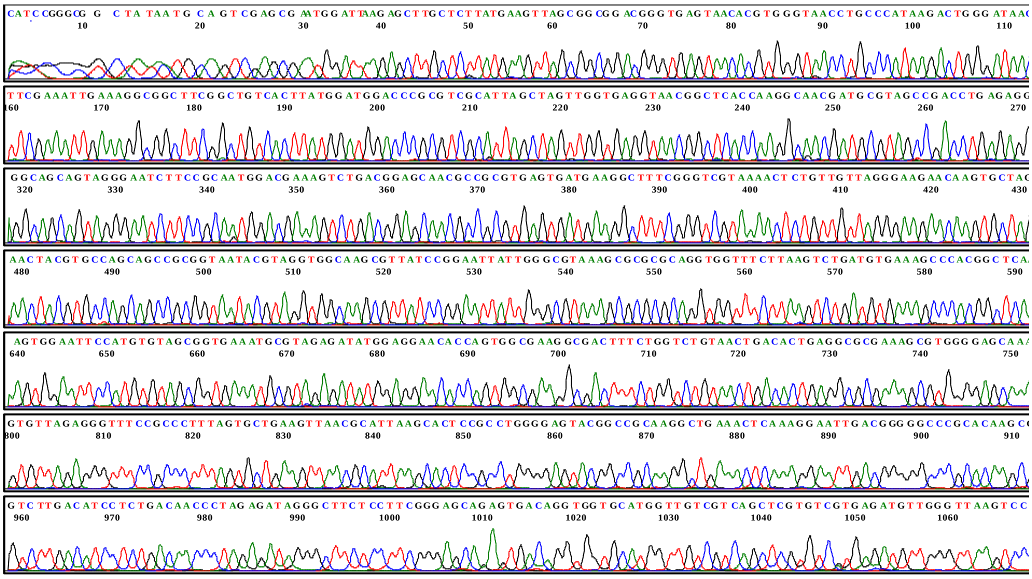

- H12

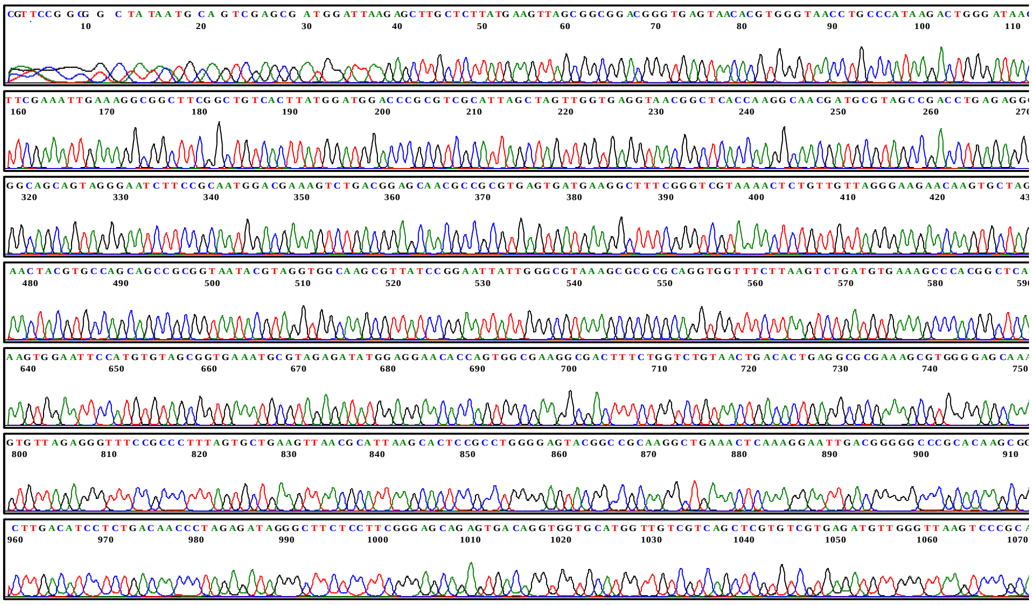

- Isolat H15

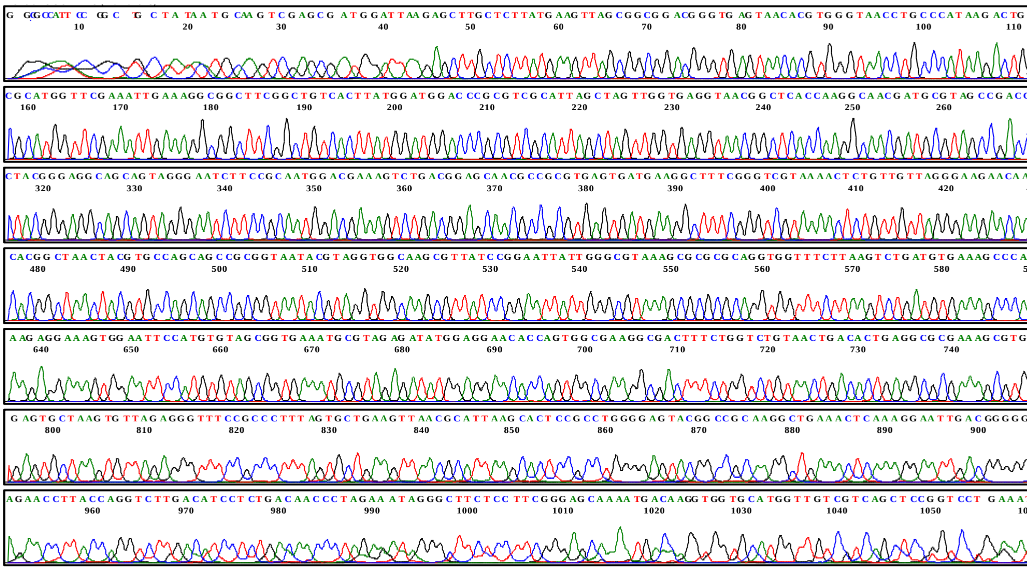

- Isolat H16

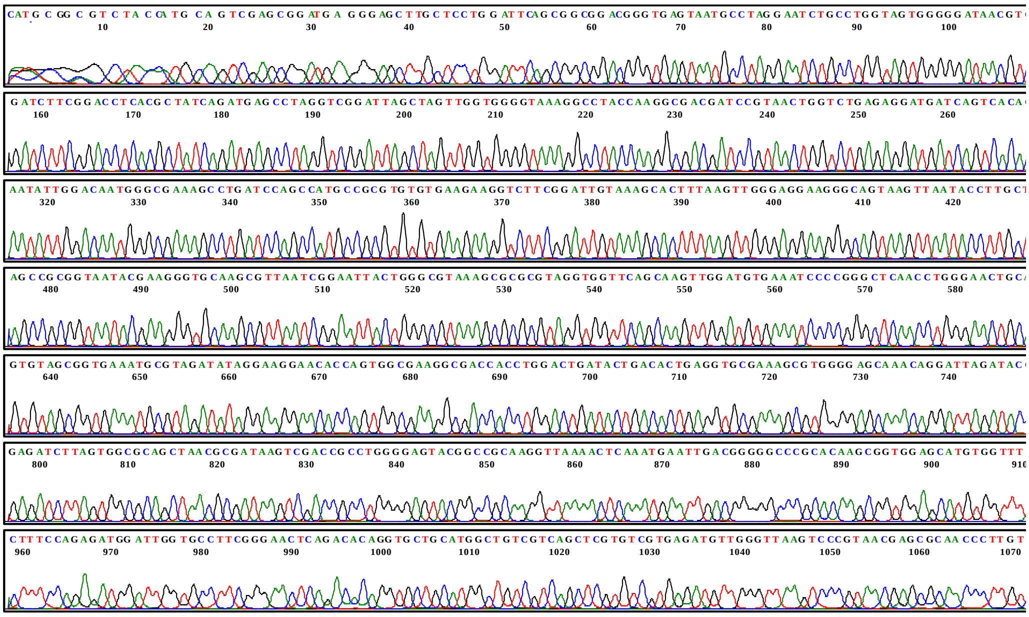

- Isolat H17

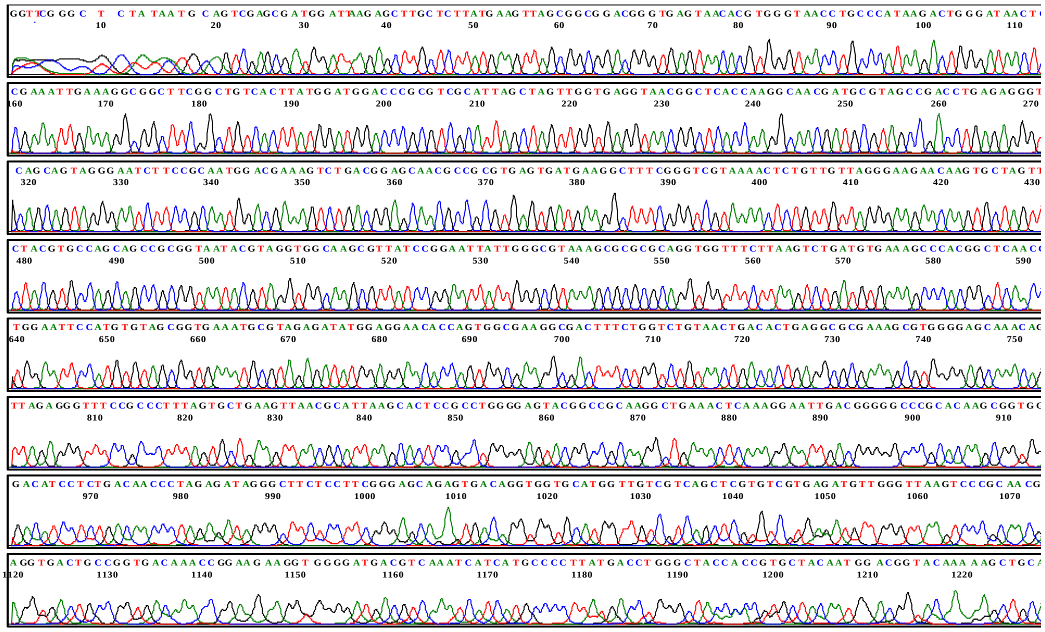

- Isolat H24

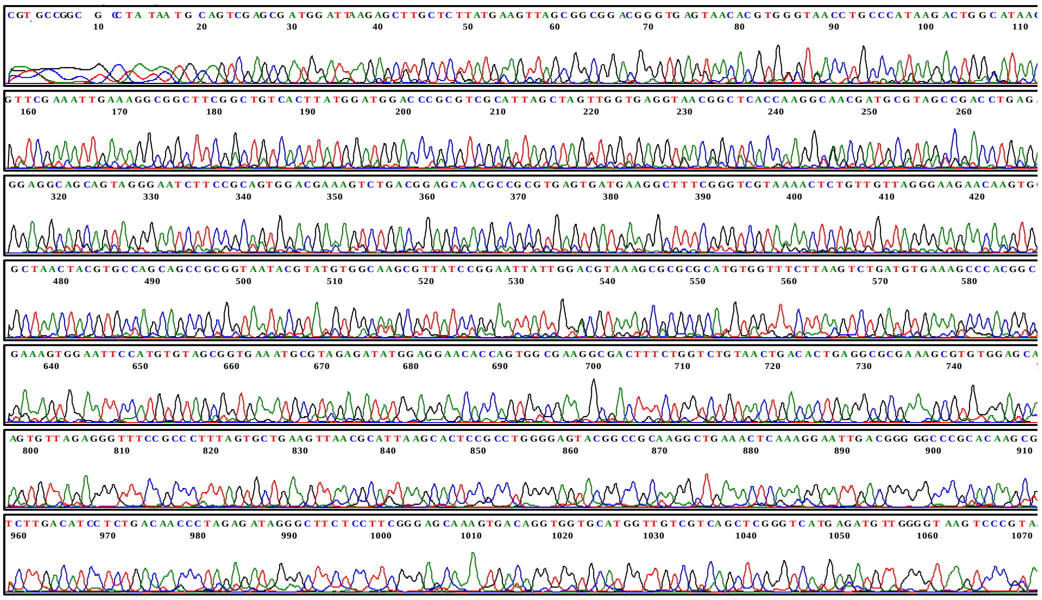

Table 2. FASTA DNA sequence of each Isolate.

Isolate	Fasta DNA
>H2 bp 1271	GGCTGAGCCGGGTCTAACATGCAGTCGAGCGAATGAATGAAGATCGCG<br>CTCGTATTCAGCTACCGACGGACGAGTGATTACCACGTGTCTGACTGGC<br>CCAGAGGAATGACATCCCTCAACGAAACCTGGTCTCATACACGACCTC<br>ATTTTGAACCTCATGGATCTTCATTCCACGGCCGCTTCGACTATCCTATA<br>TCGAAGGACCTACGTCGCGGTATCTAGGTCGAGAGGTGACAGCACACC<br>TAAGTGACCATACATAATCAACCTGCGAGGTTGATCTGACACACTGTCA<br>CTAATCCTCCGGCAAGACTCCTACGGGAAGCTTCAATAATGAATCATCC<br>CCTGTGCACGACATTCTGACTGAGCGACGACGCTCGACTGATTAAAGCT<br>TTCTTTTCGTAGAACTCTATTGTTATGGAATAACACCTTCTAGTTGAGTC<br>ATCTCCCACCTTGACGGCACCTAATCACATCGTGACAGCTAACTACGTG<br>CCACCAACCGCGCTAGTACTTATCTGACTTACTGTATCCAGAATTATTG<br>TACGTAGAGCACGCACATGTATGTTCTTACGTCTGACTCGAAATCCCAA<br>CTGTATCCGTGCAACGGCATTAGAGACTGGTAGACTTTAGTGCAATTAC<br>TATAGTGCAATGCATGTGTAGATTGAAATGCATCAACATTGGCAAGAA<br>CAACCATGTCGACTGCAACTGACTGTCTGTGCTGACACTGAGGAGCAA<br>ACCGAGTAGATACATGGATTCATACCCTGGACGACACGACTAAACGAT<br>GAATGCTAGTGTATAGTGTTCCGCCATTCGGCTGACTTCACCAGTAAGA<br>CCCCCCTGGGAGTACCTCACAAGGCTGAACTCAACGCATAACGGGGAC<br>CCGAAGCTAATACATGTACTTAATAACATAACGGCATAACTTACCAGA<br>CTTACATATATGACAGCCTCATCTAGACTTCACTTCGGATCCAGGACGC<br>TCAGCACGGTCTCACACTCGGTCAGACCGTACGTTAGTCCCTACCATAG<br>TACCTTGACTTAGTGCCACTTCAGTGACGCTCTAACTACTGCAGTAACA<br>ACCAGACACGTGCGATAAGTCAATCATCAGGCCTTATGACGGACAAGC<br>ACGTCTAAATGTACGAACAAAAGTTCAGACATCCGTGAACTATCTATA<br>AACTTTCTACTCAATTGATGCTGAGTCGCTCCTGAGCTGGATCCAAAAT<br>CCCGATCACATTCCCGGGAATAGTTCCCGGCTTTAACCTGGCGTCATCC<br>TCAAA
>H4 bp 1361	GCATTCGGGGCGCTATAATGCAAGTCGAGCGAATGGATTAAGAGCTTG<br>CTCTTATGAAGTTAGCGGCGGACGGGTGAGTAACACGTGGGTAACCTG<br>CCCATAAGACTGGGATAACTCCGGGAAACCGGGGCTAATACCGGATAA<br>CATTTTGAACCGCATGGTTCGAAATTGAAAGGCGGCTTCGGCTGTCACT<br>TATGGATGGACCCGCGTCGCATTAGCTAGTTGGTGAGGTAACGGCTCAC<br>CAAGGCAACGATGCGTAGCCGACCTGAGAGGGTGATCGGCCACACTGG<br>GACTGAGACACGGCCCAGACTCCTACGGGAGGCAGCAGTAGGGAATCT<br>TCCGCAATGGACGAAAGTCTGACGGAGCAACGCCGCGTGAGTGATGAA<br>GGCTTTCGGGTCGTAAAACTCTGTTGTTAGGGAAGAACAAGTGCTAGTT<br>GAATAAGCTGGCACCTTGACGGTACCTAACCAGAAAGCCACGGCTAAC<br>TACGTGCCAGCAGCCGCGGTAATACGTAGGTGGCAAGCGTTATCCGGA<br>ATTATTGGGCGTAAAGCGCGCGCAGGTGGTTTCTTAAGTCTGATGTGAA<br>AGCCCACGGCTCAACCGTGGAGGGTCATTGGAAACTGGGAGACTTGAG<br>TGCAGAAGAGGAAAGTGGAATTCCATGTGTAGCGGTGAAATGCGTAGA<br>GATATGGAGGAACACCAGTGGCGAAGGCGACTTTCTGGTCTGTAACTG<br>ACACTGAGGCGCGAAAGCGTGGGGAGCAAACAGGATTAGATACCCTGG<br>TAGTCCACGCCGTAAACGATGAGTGCTAAGTGTTAGAGGGTTTCCGCCC<br>TTTAGTGCTGAAGTTAACGCATTAAGCACTCCGCCTGGGGAGTACGGCC<br>GCAAGGCTGAAACTCAAAGGAATTGACGGGGGCCCGCACAAGCGGTG<br>GAGCATGTGGTTTAATTCGAAGCAACGCGAAGAACCTTACCAGGTCTT<br>GACATCCTCTGACAACCCTAGAGATAGGGCTTCTCCTTCGGGAGCAGA<br>GTGACAGGTGGTGCATGGTTGTCGTCAGCTCGTGTCGTGAGATGTTGGG<br>TTAAGTCCCGCAACGAGCGCAACCCTTGATCTTAGTTGCCATCATTCAG<br>TTGGGCACTCTAAGGTGACTGCCGGTGACAAACCGGAGGAAGGTGGGG<br>ATGACGTCAAATCATCATGCCCCTTATGACCTGGGCTACCCACGTGCTA<br>CAATGGACGGTACCAAAGAGCTGCAAGACCGCCGAGGTGGAACCTAAT<br>CTCATAAAACCCGTTCTCAGTTCCGAATTGAAGGCTGGAACTCCGCCTA<br>CATGAAACTTGGAATCGCTAAGTAATCCCCGAATCAACCTTGCCCCCGG<br>TGGATAC
>H8 bp 1240	CCTGCCGGAGCTACCATGCAGTCGAGCGGATGAGGGAGCTTGCTCCTG<br>GATTCAGCGGCGGACGGGTGAGTAATGCCTAGGAATCTGCCTGGTAGT<br>GGGGGATAACGTCCGGAAACGGGCGCTAATACCGCATACGTCCTGAGG<br>GAGAAAGTGGGGGATCTTCGGACCTCACGCTATCAGATGAGCCTAGGT<br>CGGATTAGCTAGTTGGTGGGGTAAAGGCCTACCAAGGCGACGATCCGT<br>AACTGGTCTGAGAGGATGATCAGTCACACTGGAACTGAGACACGGTCC

	AGACTCCTACGGGAGGCAGCAGTGGGGAATATTGGACAATGGGCGAAA<br>GCCTGATCCAGCCATGCCGCGTGTGTGAAGAAGGTCTTCGGATTGTAAA<br>GCACTTTAAGTTGGGAGGAAGGGCAGTAAGTTAATACCTTGCTGTTTTG<br>ACGTTACCAACAGAATAAGCACCGGCTAACTTCGTGCCAGCAGCCGCG<br>GTAATACGAAGGGTGCAAGCGTTAATCGGAATTACTGGGCGTAAAGCG<br>CGCGTAGGTGGTTCAGCAAGTTGGATGTGAAATCCCCGGGCTCAACCT<br>GGGAACTGCATCCAAAACTACTGAGCTAGAGTACGGTAGAGGGTGGTG<br>GAATTTCCTAACTCAAATGAATTGACGGGGGCCCGCACAAGCGGTGGA<br>GCATGTGGTTTAATTCGAAGCAACGCGAAGAACCTTACCTGGCCTTGAC<br>ATGCTGAGAACTTTCCAGAGATGGATTGGTGCCTTCGGGAACTCAGAC<br>ACAGGTGCTGCATGGCTGTCGTCAGCTCGTGTCGTGAGATGTTGGGTTA<br>AGTCCCGTAACGAGCGCAACCCTTGTCCTTAGTTACCAGCACCTCGGGT<br>GGGCACTCTAAGGAGACTGCCGGTGACAAACCGGAGGAAGGTGGGGA<br>TGACGTCAAGTCATCATGGCCCTTACGGCCAGGGCTACACACGTGCTAC<br>AATGGTCGGTACAAAGGGTTGCCAAGCCGCGAGGTGGAGCTAATCCCA<br>TAAAACCGATCGTAGTCCGGATCGCAGTCTGCAACTCGACTGCGTGAA<br>GTCGGAATCGCTAGTAATCGTGAATCAGAATGTCACGGTGAATACGTTC<br>CCGGGCCTTGTACACACCGCCCGTCACACCATGGGAGTGGGTTGCTCCA<br>GAAGTAGCTAGTCTAACCGCAAGGGGGACGGTTACCACGGAGTGATTC<br>ATGACTGGGGTGAAGTCGTATAAGAGCCTCGCC
>H9 bp 1160	GGGGGTTAATTCCGGGATAACATTTTGAACCCCCATGGTTCGAAATTGA<br>AAGGCGGCTTCGGCTGTCACTTATGGATGGACCCGCGTCGCATTAGCTA<br>GTTGGTGAGGTAACGGCTCACCAAGGCAACGATGCGTAGCCGACCTGA<br>GAGGGTGATCGGCCACACTGGGACTGAGACACGGCCCAGACTCCTACG<br>GGAGGCAGCAGTAGGGAATCTTCCGCAATGGACGAAAGTCTGACGGAG<br>CAACGCCGCGTGAGTGATGAAGGCTTTCGGGTCGTAAAACTCTGTTGTT<br>AGGGAAGAACAAGTGCTAGTTGAATAAGCTGGCACCTTGACGGTACCT<br>AACCAGAAAGCCACGGCTAACTACGTGCCAGCAGCCGCGGTAATACGT<br>AGGTGGCAAGCGTTATCCGGAATTATTGGGCGTAAAGCGCGCGCAGGT<br>GGTTTCTTAAGTCTGATGTGAAAGCCCACGGCTCAACCGTGGAGGGTCA<br>TTGGAAACTGGGAGACTTGAGTGCAGAAGAGGAAAGTGGAATTCCATG<br>TGTAGCGGTGAAATGCGTAGAGATATGGAGGAACACCAGTGGCGAAGG<br>CGACTTTCTGGTCTGTAACTGACACTGAGGCGCGAAAGCGTGGGGAGC<br>AAACAGGATTAGATACCCTGGTAGTCCACGCCGTAAACGATGAGTGCT<br>AAGTGTTAGAGGGTTTCCGCCCTTTAGTGCTGAAGTTAACGCATTAAGC<br>ACTCCGCCTGGGGAGTACGGCCGCAAGGCTGAAACTCAAAGGAATTGA<br>CGGGGGCCCGCACAAGCGGTGGAGCATGTGGTTTAATTCGAAGCAACG<br>CGAAGAACCTTACCAGGTCTTGACATCCTCTGAAAACCCTAGAGATAG<br>GGCTTCTCCTTCGGGAGCAGAGTGACAGGTGGTGCATGGTTGTCGTCAG<br>CTCGTGTCGTGAGATGTTGGGTTAAGTCCCGCAACGAGCGCAACCCTTG<br>ATCTTAGTTGCCATCATTAAGTTGGGCACTCTAAGGTGACTGCCGGTGA<br>CAAACCGGAGGAAGGTGGGGATGACGTCAAATCATCATGCCCCTTATG<br>ACCTGGGCTACACACGTGCTACAATGGACGGTACAAAGAGCTGCAAGA<br>CCGCGAGGTGGAGCTAATCTCATAAAACCGTTCTCAGTTCGGATTGTA
>H10 bp 1278	GCCGCCAAAGACTGGAAATCGGGAACCGGGTTATCCGGATACATTTGA<br>CCGCTGGTCGAAATGAAGGCGCTTGGCTTCATTAGGATGACCGGGTCC<br>ATTACTAGTGGTGGGTACCGCTCCCAAGCACGATCGTACCGCCTGAAG<br>GGGATGGCCCCCTGGATGAGCCCGGCCAGATCCTACGGGAGCAGCGAG<br>GGATCTTCGCAATGACGAAAGTCTGACGAGCAAGCCGCTGATGAAGAA<br>GCTTTCGGTCGTAAACTCGTTGTTAGGAAGACCAATGCTAGTGAATAGC<br>TGGCACTTGACGGACCTACCAGAAGCCCGGCTAATTCGTGCAGCAGCG<br>CGGTAATCGTAGGGGCAACGTTATCGGAATTATGGCGTAAGCGCCGCA<br>GTGGTTTCTAAGTCTGATGGAAAGCCCAGGCTCACCGGTGAGGTCATG<br>AAACTGGGGACTGAGTGCAAAGAGAAAGTGAATTCCAGGGTAGCGGG<br>AAATGCGTAAGATAGGAGAACACCAGTGCGAAGCGACTTCTGGTCTGT<br>AATGACACGAGGGCGAAAGCGTGGGAGCAACAGGATAGATACCCGGT<br>AGTCCAGCCGTAACGATGAGTGCTAAGGTTAGAGGGTTTCCGCCCTTTA<br>GTGCTGAAGTAACGCATTAAGCACTCCGCCTGGGGAGTACGGCCGCAA<br>GGCTGAAACTCAAAGGAATTGACGGGGGCCCGCACAAGCGGTGGAGC<br>ATGTGGTTTAATTGGAAGCAACGCGAAGAACCTTACCAGGTCTTGACAT<br>CTTCTGCAACCCTAGAGATAGGGCTTCTCCTTCGGGAGCAGAGTGACAG<br>GTGGTGCATGGTTGTCGTCAGCTCGTGTCGTGAGATGTTGGGTTAAGTC<br>CCGCAACGAGCGCAACCCTTGATCTTAGTTGCCATCATTCAGTTGGCCA<br>CTCTAAGGTGACTGCCGGTGACAAACCGGAGGAAGGTGGGGATGACGT<br>CAAATCATCATGCCCCTTATGACCTGGGCTACACACGTGCTCCAATGGA<br>CGGTACAAAGAGCTGCAAGACCGCGAGGTGGAGCTAATCTCATAAAAC<br>CGTTCTCAGTTCGGATTGTAGGCTGCAACTCGCCTACATGAAGCTGGAA

	TCGCTAGTAATCGCGGATCAGCATGCCGCGGTGAATACGTTCCCGGGCC<br>TTGTACACACCGCCCGTCACACCACGAGAGTTTGTAACACCCGAAGTCG<br>GTGGGGTAACCTTTTGGAGCCAGCCGCCTAAGGTGGGACAGATGATGG<br>GGTGAAGTCGTAACAAGTGCTGC
>H12 bp 1360	CGTTCCGGCGGCTATAATGCAGTCGAGCGATGGATTAAGAGCTTGCTCT<br>TATGAAGTTAGCGGCGGACGGGTGAGTAACACGTGGGTAACCTGCCCA<br>TAAGACTGGGATAACTCCGGGAAACCGGGGCTAATACCGGATAACATT<br>TTGAACCGCATGGTTCGAAATTGAAAGGCGGCTTCGGCTGTCACTTATG<br>GATGGACCCGCGTCGCATTAGCTAGTTGGTGAGGTAACGGCTCACCAA<br>GGCAACGATGCGTAGCCGACCTGAGAGGGTGATCGGCCACACTGGGAC<br>TGAGACACGGCCCAGACTCCTACGGGAGGCAGCAGTAGGGAATCTTCC<br>GCAATGGACGAAAGTCTGACGGAGCAACGCCGCGTGAGTGATGAAGGC<br>TTTCGGGTCGTAAAACTCTGTTGTTAGGGAAGAACAAGTGCTAGTTGAA<br>TAAGCTGGCACCTTGACGGTACCTAACCAGAAAGCCACGGCTAACTAC<br>GTGCCAGCAGCCGCGGTAATACGTAGGTGGCAAGCGTTATCCGGAATT<br>ATTGGGCGTAAAGCGCGCGCAGGTGGTTTCTTAAGTCTGATGTGAAAG<br>CCCACGGCTCAACCGTGGAGGGTCATTGGAAACTGGGAGACTTGAGTG<br>CAGAAGAGGAAAGTGGAATTCCATGTGTAGCGGTGAAATGCGTAGAGA<br>TATGGAGGAACACCAGTGGCGAAGGCGACTTTCTGGTCTGTAACTGAC<br>ACTGAGGCGCGAAAGCGTGGGGAGCAAACAGGATTAGATACCCTGGTA<br>GTCCACGCCGTAAACGATGAGTGCTAAGTGTTAGAGGGTTTCCGCCCTT<br>TAGTGCTGAAGTTAACGCATTAAGCACTCCGCCTGGGGAGTACGGCCG<br>CAAGGCTGAAACTCAAAGGAATTGACGGGGGCCCGCACAAGCGGTGG<br>AGCATGTGGTTTAATTCGAAGCAACGCGAAGAACCTTACCAGGTCTTG<br>ACATCCTCTGACAACCCTAGAGATAGGGCTTCTCCTTCGGGAGCAGAGT<br>GACAGGTGGTGCATGGTTGTCGTCAGCTCGTGTCGTGAGATGTTGGGTT<br>AAGTCCCGCAACGAGCGCAACCCTTGATCTTAGTTGCCATCATTCAGTT<br>GGGCACTCTAAGGTGACTGCCGGTGACAAACCGGAGGAAGGTGGGGAT<br>GACGTCAAATCATCATGCCCCTTATGACCTGGGCTACACACGTGCTACA<br>ATGGACGGTACAAAGAGCTGCAAGACCGCCAGGTGGAGCTAATCTCAT<br>AAAACCGTTCTCAGTTCGGATTGTAGGCTGCAACTCGCCTACATGAAGC<br>TGGAATCGCTAGTAATCGCGGATCAGCATGGCCCCGGTGAATACGTTTC<br>CCGGGCA
>H15 bp 1253	GCTCTTATGAAGTTAGCGGCGGACGGGTGAGTAACACGTGGGTAACCT<br>GCCCATAAGACTGGGATAACTCCGGGAAACCGGGGCTAATACCGGATA<br>ACATTTTGAACCGCATGGTTCGAAATTGAAAGGCGGCTTCGGCTGTCAC<br>TTATGGATGGACCCGCGTCGCATTAGCTAGTTGGTGAGGTAACGGCTCA<br>CCAAGGCAACGATGCGTAGCCGACCTGAGAGGGTGATCGGCCACACTG<br>GGACTGAGACACGGCCCAGACTCCTACGGGAGGCAGCAGTAGGGAATC<br>TTCCGCAATGGACGAAAGTCTGACGGAGCAACGCCGCGTGAGTGATGA<br>AGGCTTTCGGGTCGTAAAACTCTGTTGTTAGGGAAGAACAAGTGCTAGT<br>TGAATAAGCTGGCACCTTGACGGTACCTAACCAGAAAGCCACGGCTAA<br>CTACGTGCCAGCAGCCGCGGTAATACGTAGGTGGCAAGCGTTATCCGG<br>AATTATTGGGCGTAAAGCGCGCGCAGGTGGTTTCTTAAGTCTGATGTGA<br>AAGCCCACGGCTCAACCGTGGAGGGTCATTGGAAACTGGGAGACTTGA<br>GTGCAGAAGAGGAAAGTGGAATTCCATGTGTAGCGGTGAAATGCGTAG<br>AGATATGGAGGAACACCAGTGGCGAAGGCGACTTTCTGGTCTGTAACT<br>GACACTGAGGCGCGAAAGCGTGGGGAGCAAACAGGATTAGATACCCTG<br>GTAGTCCACGCCGTAAACGATGAGTGCTAAGTGTTAGAGGGTTTCCGCC<br>CTTTAGTGCTGAAGTTAACGCATTAAGCACTCCGCCTGGGGAGTACGGC<br>CGCAAGGCTGAAACTCAAAGGAATTGACGGGGGCCCGCACAAGCGGTG<br>GAGCATGTGGTTTAATTCGAAGCAACGCGAAGAACCTTACCAGGTCTT<br>GACATCCTCTGACAACCCTAGAAATAGGGCTTCTCCTTCGGGAGCAAA<br>ATGACAAGGTGGTGCATGGTTGTCGTCAGCTCCGGTCCTGAAATGTTGG<br>GTTAAGTCCCGCAACGAGCGCAACCCTTGATCTTAGTTGCCATCATTCA<br>GTTGGGCACCCTAAGGTGACTGCCGGGTGACAAACCGGAAGGAAGGTG<br>GGGGATGAACGTCAAATCCTCATGGCCCCTAATGGACCTGGGCCTACC<br>ACCGTGCTACAATGGAACGGTACAAAAGACTTCCAAAAACCGCCAGGT<br>GGAACTTAATTCCCAAAAAACCCGTTCCCAGGTTCGGAATTGGAA
>H16 bp 927	ACCGCCTGGGGAGTACGGCCGCAAGGTTAAAACTCAAATGAATTGACG<br>GGGGCCCGCACAAGCGGTGGAGCATGTGGTTTAATTCGAAGCAACGCG<br>AAGAACCTTACCTGGCCTTGACATGCTGAGAACTTTCCAGAGATGGATT<br>GGTGCCTTCGGGAACTCAGACACAGGTGCTGCATGGCTGTCGTCAGCTC<br>GTGTCGTGAGATGTTGGGTTAAGTCCCGTAACGAGCGCAACCCTTGTCC<br>TTAGTTACCAGCACCTCGGGTGGGCACTCTAAGGAGACTGCCGGTGAC<br>AAACCGGAGGAAGGTGGGGATGACGTCAAGTCATCATGGCCCTTACGG<br>CCAGGGCTACACACGTGCTACAATGGTCGGTACAAAGGGTTGCCAAGC

	CGCGAGGTGGAGCTAATCCCATAAAACCGATCGTAGTCCGGATCGCAG<br>TCTGCAACTCGACTGCGTGAAGTCGGAATCGCTAGTAATCGTGAATCAA<br>AATGTCACGGTGAAACTGGGTTAAGTCCCGTAACGAGCGCAACCCTTG<br>TCCTTAGTTACCAGCACCTCGGGTGGGCACTCTAAGGAGACTGCCGGTG<br>ACAAACCGGAGGAAGGTGGGGATGACGTCAAGTCATCATGGCCCTTAC<br>GGCCAGGGCTACACACGTGCTACAATGGTCGGTACAAAGGGTTGCCAA<br>GCCGCGAGGTGGAGCTAATCCCATAAAACCGATCGTAGTCCGGATCGC<br>AGTCTGCAACTCGACTGCGTGAAGTCGGAATCGCTAGTAATCGTGAATC<br>AGAATGTCACGGTGAATACGTTCCCGGGCCTTGTACACACCGCCCGTCA<br>CACCATGGGAGTGGGTTGCTCCAGAAGTAGCTAGTCTAACCGCAAGGG<br>GGACGGTTACCACGGAGTGATTCATGACTGGGGTGAAGTCGTAACAAG<br>TGCCTGCC
>H17 bp 1195	TGAAGTTAGCGGCGGACGGGTGAGTAACACGTGGGTAACCTGCCCATA<br>AGACTGGGATAACTCCGGGAAACCGGGGCTAATACCGGATAACATTTT<br>GAACCGCATGGTTCGAAATTGAAAGGCGGCTTCGGCTGTCACTTATGG<br>ATGGACCCGCGTCGCATTAGCTAGTTGGTGAGGTAACGGCTCACCAAG<br>GCAACGATGCGTAGCCGACCTGAGAGGGTGATCGGCCACACTGGGACT<br>GAGACACGGCCCAGACTCCTACGGGAGGCAGCAGTAGGGAATCTTCCG<br>CAATGGACGAAAGTCTGACGGAGCAACGCCGCGTGAGTGATGAAGGCT<br>TTCGGGTCGTAAAACTCTGTTGTTAGGGAAGAACAAGTGCTAGTTGAAT<br>AAGCTGGCACCTTGACGGTACCTAACCAGAAAGCCACGGCTAACTACG<br>TGCCAGCAGCCGCGGTAATACGTAGGTGGCAAGCGTTATCCGGAATTA<br>TTGGGCGTAAAGCGCGCGCAGGTGGTTTCTTAAGTCTGATGTGAAAGCC<br>CACGGCTCAACCGTGGAGGGTCATTGGAAACTGGGAGACTTGAGTGCA<br>GAAGAGGAAAGTGGAATTCCATGTGTAGCGGTGAAATGCGTAGAGATA<br>TGGAGGAACACCAGTGGCGAAGGCGACTTTCTGGTCTGTAACTGACAC<br>TGAGGCGCGAAAGCGTGGGGAGCAAACAGGATTAGATACCCTGGTAGT<br>CCACGCCGTAAACGATGAGTGCTAAGTGTTAGAGGGTTTCCGCCCTTTA<br>GTGCTGAAGTTAACGCATTAAGCACTCCGCCTGGGGAGTACGGCCGCA<br>AGGCTGAAACTCAAAGGAATTGACGGGGGCCCGCACAAGCGGTGGAG<br>CATGTGGTTTAATTCGAAGCAACGCGAAGAACCTTACCAGGTCTTGACA<br>TCCTCTGACAACCCTAGAGATAGGGCTTCTCCTTCGGGAGCAGAGTGAC<br>AGGTGGTGCATGGTTGTCGTCAGCTCGTGTCGTGAGATGTTGGGTTAAG<br>TCCCGCAACGAGCGCAACCCTTGATCTTAGTTGCCATCATTCAGTTGGG<br>CACTCTAAGGTGACTGCCGGTGACAAACCGGAAGAAGGTGGGGATGAC<br>GTCAAATCATCATGCCCCTTATGACCTGGGCTACCACCGTGCTACAATG<br>GACGGTACAAAAAGCTGCAAAACCCCCAGGTGGAGC
>H24 bp 1391	CGTGCCGGCGCCTATAATGCAGTCGAGCGATGGATTAAGAGCTTGCTCT<br>TATGAAGTTAGCGGCGGACGGGTGAGTAACACGTGGGTAACCTGCCCA<br>TAAGACTGGCATAACTCCGGGAAACCGGGGCTAATACCGGATAACATT<br>TTGAACCGCATGGTTCGAAATTGAAAGGCGGCTTCGGCTGTCACTTATG<br>GATGGACCCGCGTCGCATTAGCTAGTTGGTGAGGTAACGGCTCACCAA<br>GGCAACGATGCGTAGCCGACCTGAGAGGGTGATCGGCCACACTGGGAC<br>TGAGACACGGCCCAGACTCCTACGGGAGGCAGCAGTAGGGAATCTTCC<br>GCAGTGGACGAAAGTCTGACGGAGCAACGCCGCGTGAGTGATGAAGGC<br>TTTCGGGTCGTAAAACTCTGTTGTTAGGGAAGAACAAGTGCTAGTTGAA<br>TAAGCTGGCACCTTGACGGTACCTAACCAGAAAGCCACGGCTAACTAC<br>GTGCCAGCAGCCGCGGTAATACGTATGTGGCAAGCGTTATCCGGAATT<br>ATTGGACGTAAAGCGCGCGCATGTGGTTTCTTAAGTCTGATGTGAAAGC<br>CCACGGCTCAACCGTGGAGGGTCATTGGAAACTGGGAGACTTGAGTGC<br>AGAAGAGGAAAGTGGAATTCCATGTGTAGCGGTGAAATGCGTAGAGAT<br>ATGGAGGAACACCAGTGGCGAAGGCGACTTTCTGGTCTGTAACTGACA<br>CTGAGGCGCGAAAGCGTGTGGAGCAAACAGGATTAGATACCCTGGTAG<br>TCCACGCCGTAAACGATGAGTGCTAAGTGTTAGAGGGTTTCCGCCCTTT<br>AGTGCTGAAGTTAACGCATTAAGCACTCCGCCTGGGGAGTACGGCCGC<br>AAGGCTGAAACTCAAAGGAATTGACGGGGGCCCGCACAAGCGGTGGA<br>GCATGTGGTTTAATTCGAAGCAACGCGAATAACCTTACCAGGTCTTGAC<br>ATCCTCTGACAACCCTAGAGATAGGGCTTCTCCTTCGGGAGCAAAGTGA<br>CAGGTGGTGCATGGTTGTCGTCAGCTCGGGTCATGAGATGTTGGGGTAA<br>GTCCCGTAACGAGCGCAACCCTTGATCTTAGTTGCCATCATTCAGTTGG<br>GCACTCTAAGGTAACTGCCGGTGACAAACCGGAAGAAGGTGGGGATGA<br>CGTCAAATCATCATGGCCCTTATGACCTGGGCTACGACCGTGCTACAAT<br>GTACGGTACAAAAAGCTGCAAGACCGCCAGGTGGAACTAATCTCATAA<br>AACCGTTCTCAGTTCGAATTGAAGGCTGCAACTCGCCTACCTGAAGCTT<br>GAATCGCTAAGTATCCCGGATCAACATGCCCCGGGGGAATACGTTTCCC<br>GGGGCTTTGAACAACCGGCCCGTTCAACCCCCGAGC
